# Allele-specific expression and alternative splicing in horse×donkey and cattle×yak hybrids

**DOI:** 10.24272/j.issn.2095-8137.2019.042

**Published:** 2019-07-18

**Authors:** Yu Wang, Shan Gao, Yue Zhao, Wei-Huang Chen, Jun-Jie Shao, Ni-Ni Wang, Ming Li, Guang-Xian Zhou, Lei Wang, Wen-Jing Shen, Jing-Tao Xu, Wei-Dong Deng, Wen Wang, Yu-Lin Chen, Yu Jiang

**Affiliations:** 1 Key Laboratory of Animal Genetics, Breeding and Reproduction of Shaanxi Province, College of Animal Science and Technology, Northwest A&F University, Yangling Shaanxi 712100, China; 2 Stake Key Laboratory of Plateau Ecology and Agriculture, Qinghai Academy of Animal Science and Veterinary Medicine, Qinghai University, Xining Qinghai 810016, China; 3 State Key Laboratory of Genetic Resources and Evolution, Kunming Institute of Zoology, Chinese Academy of Sciences, Kunming Yunnan 650223, China; 4 Faculty of Animal Science and Technology, Yunnan Agricultural University, Kunming Yunnan 650223, China

**Keywords:** Allele-specific alternative splicing, Allele-specific expression, *Cis*-regulatory elements, Hybrid species

## Abstract

Divergence of gene expression and alternative splicing is a crucial driving force in the evolution of species; to date, however the molecular mechanism remains unclear. Hybrids of closely related species provide a suitable model to analyze allele-specific expression (ASE) and allele-specific alternative splicing (ASS). Analysis of ASE and ASS can uncover the differences in *cis*-regulatory elements between closely related species, while eliminating interference of *trans-*regulatory elements. Here, we provide a detailed characterization of ASE and ASS from 19 and 10 transcriptome datasets across five tissues from reciprocal-cross hybrids of horse×donkey (mule/hinny) and cattle×yak (dzo), respectively. Results showed that 4.8%–8.7% and 10.8%–16.7% of genes exhibited ASE and ASS, respectively. Notably, lncRNAs and pseudogenes were more likely to show ASE than protein-coding genes. In addition, genes showing ASE and ASS in mule/hinny were found to be involved in the regulation of muscle strength, whereas those of dzo were involved in high-altitude adaptation. In conclusion, our study demonstrated that exploration of genes showing ASE and ASS in hybrids of closely related species is feasible for species evolution research.

## INTRODUCTION

The accumulation of genetic variations in a genome sequence results in phenotypic diversity and adaptive evolution, with the majority of genetic variations functioning in gene expression regulation (Keane et al., 2011; Kwan et al., 2008). Therefore, identification of changes in the gene expression profiles, including expression levels and alternative splicing, between closely related species (e.g., horse and donkey, cattle and yak) could help clarify the genetic basis of species adaptive evolution. However, it is widely accepted that environmental factors can also affect gene expression (Forrest et al., 2014; Prabhakar et al., 2008; Villar et al., 2015), which can hinder comparisons of gene expression profiles between species (Brown et al., 2014).

Hybrids of closely related species provide a good model for interspecific comparisons of gene expression proﬁles at the allelic level (Tirosh et al., 2009). The relative expression profiles of two alleles of a heterozygous variant can be assessed by allele-specific expression (ASE) and allele-specific splicing (ASS). To date, most studies on ASE and ASS genes have been primarily identified in model organisms, such as the mouse (Eckersley-Maslin & Spector, 2014; Pinter et al., 2015; Wood et al., 2015). Crowley et al.(2015)used highly divergent mouse crosses to analyze ASE and found that more than 80% of genes exhibited *cis*-regulatory variation, thus suggesting that pervasive gene expression regulatory variation can influence complex genetic traits and thereby contributed to the adaptive evolution of mice. In addition, ASS has also been identified using hybrids of divergent C57BL/6J and SPRET/EiJ mouse strains, which showed that *cis*-regulatory changes resulted in alternative splicing in the evolution of mice (Gao et al., 2015). In the current study, we expanded ASE and ASS research to large mammals (horse×donkey and cattle×yak hybrids) and explored changes in gene expression in regard to adaptive evolution.

Horse (*Equus caballus*) and donkeys (*Equus asinus*) are domesticated members of *Equus* that diverged approximately 4.0–4.5 million years ago (Orlando et al., 2013). The main difference between these species is in muscle strength, with horses exhibiting greater initial power over short distances and donkeys showing greater stamina over longer distances. Cattle (*Bos taurus*) and yaks (*Bos grunniens*) are members of the bovine family and diverged approximately 4.9 million years ago (Qiu et al., 2012). The most remarkable difference between these species is the ability of yaks to adapt to high-altitude environments. However, the molecular bases of the adaptive evolution between the above closely related species remain unclear.

**Figure 1:**
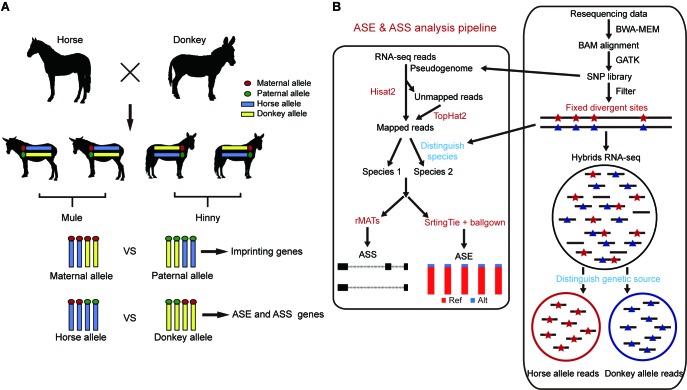
Pipeline for ASE and ASS analysis Resequencing data were mapped to the genome using BWA. Divergent site calling was then performed by GATK. The pseudogenome was constructed. Divergent sites were filtered by RNA-seq data and used to calculate ASE. Hybrid transcriptomes were divided into two genetic allelic samples using fixed divergent sites. Separated genetic allelic samples were used to calculate ASS. rMATS: Replicate multivariate analysis of transcript splicing. Ref: Reference allele; Alt: Alternative allele.

In this study, we used reciprocal-cross hybrids of horse×donkey and cattle×yak to calculate ASE and imprinting genes and to explore the evolution of *cis*-regulatory gene expression and alternative splicing (Figure 1). In addition, to elucidate fixed expression changes between closely related species, those genes showing ASE in all biological replicates were retained and individually specific ASE genes were filtered. Our results demonstrated that both gene expression and alternative splicing contributed to the divergence between closely related species. Furthermore, genes showing ASE and ASS participated in the regulation of phenotype differences, including muscle strength in mule/hinny and high-altitude adaptation in dzo.

## MATERIALS AND METHODS

### Samples

**Figure 2:**
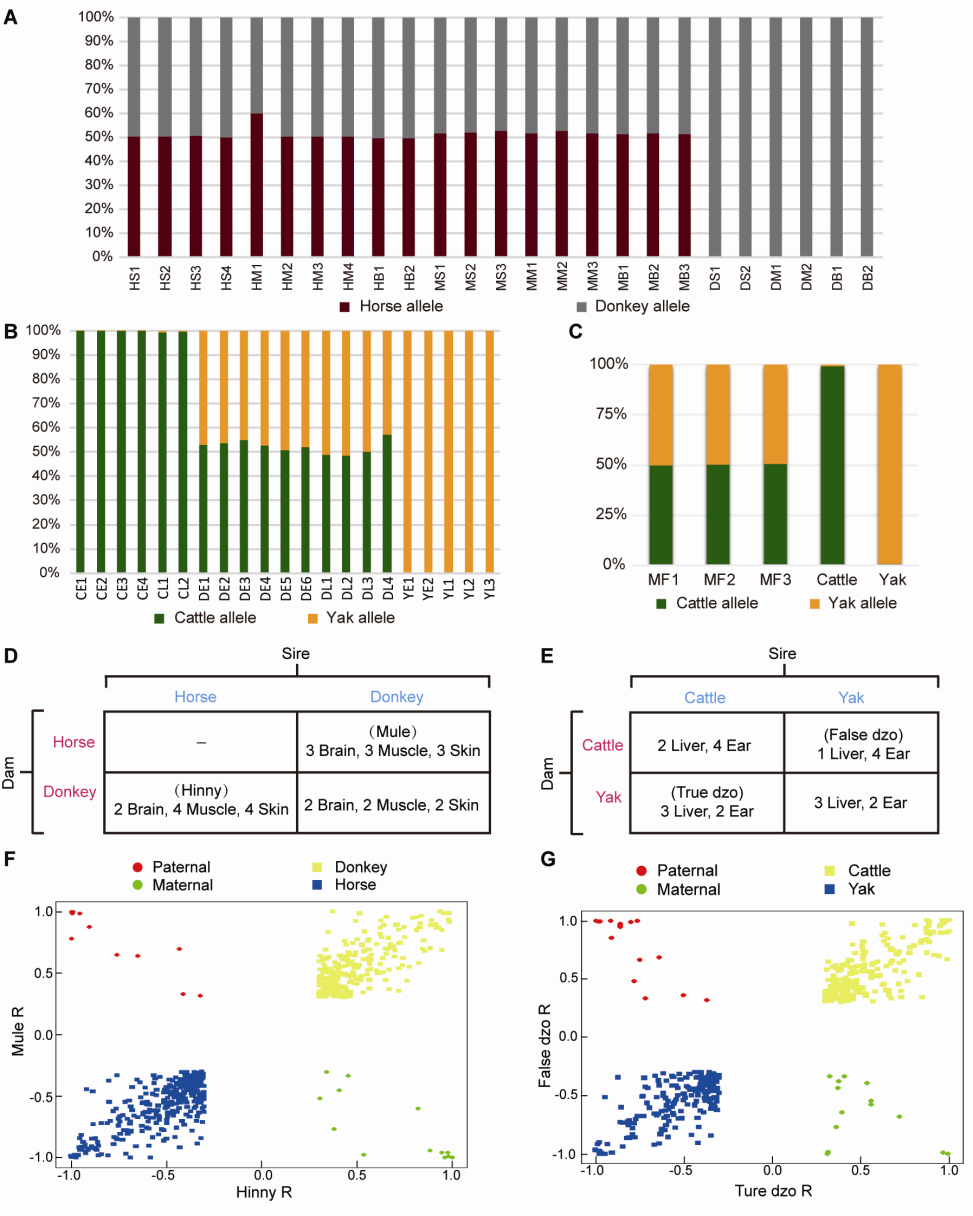
Reciprocal-cross hybrid samples to identify ASE A: RNA-seq paired-end reads of mule/hinny and donkey were assigned a genetic allele origin. Proportions of the horse and donkey allele origins are shown. HS: Hinny skin tissue; HM: Hinny muscle tissue; HB: Hinny brain tissue; MS: Mule skin tissue; MM: Mule muscle tissue; MB: Mule brain tissue; DS: Donkey skin tissue; DM: Donkey muscle tissue; DB: Donkey brain tissue. B: RNA-seq reads of cattle, yak, and dzo were assigned a genetic allele origin. Proportions of cattle and yak allele origin are shown. CE: Cattle ear tissue; CL: Cattle liver tissue; DE: Dzo ear tissue; DL: Dzo liver tissue; YE: Yak ear tissue; YL: Yak liver tissue. C: Mock hybrid transcriptomes were assigned a genetic allele origin. MF: Mock hybrids. D: Sample and diallel crossing scheme of horse and donkey, mule (female horse×male donkey), and hinny (male horse×female donkey). E: Sample and diallel crossing scheme of cattle and yak, true dzo (male cattle×female yak), and false dzo (female cattle×male yak). F: Distribution of skew (*R*) values (where read ratio *R*=(horse-donkey)/(horse+donkey)) from -1 to 1 for genes showing ASE (yellow, blue) and imprinting genes (red, green) in mule and hinny brain tissues. G: Distribution of skew (*R*) values in true and false dzo ear tissues (*R*= (cattle-yak)/(cattle+yak)).

Samples from hybrids of horse and donkey (10-year-old mules/hinnies) were obtained from Yulin City, Shaanxi Province (see Supplementary Table S1 for detailed sample information). Samples from hybrids of cattle and yak (two- to five-year-old dzos) were obtained from Xining City, Qinghai Province (ear tissue) and Diqing City, Yunnan Province (liver tissue) (see Supplementary Table S2 for detailed sample information). The skin (back of neck, *n*=7, three mule and four hinny samples), brain (prefrontal lobe, *n*=5, three mule and two hinny samples), muscle (semitendinosus muscle, *n*=7, three mule and four hinny samples), liver (*n*=4, three true and one false dzo samples), and ear (*n*=6, two true and four false dzo samples) were dissected and rinsed with PBS. In addition, two skin, two brain, and two muscle tissue samples from donkeys, two liver and four ear tissue samples from cattle, and three liver and two ear tissue samples from yaks were also dissected for RNA sequencing (RNA-seq) (Figure 2D, E). The samples were frozen in liquid nitrogen and stored at –80 °C freezer until required. The study was approved by the Institutional Animal Care and Use Committee of Northwest A&F University (Permit Number: NWAFAC1019).

### RNA-seq library construction and sequencing

Total RNA from the frozen samples was extracted using TRIzol reagent (Invitrogen, USA) following the protocols stated by the manufacturer. Genomic DNA contamination was first removed using RNA-free DNase I, and RNA integrity and quality were then analyzed using a bioanalyzer (Agilent, USA). The RNA integrity threshold was RIN≥6.8. The PolyA(+) RNA-seq libraries were constructed using a NEBNext® Ultra^TM^ RNA Library Prep Kit for Illumina® (NEB, USA) according to the manufacturer’s recommendations. The resulting cDNA was first cleaved into 300–500 bp fragments to construct libraries according to the manufacturer‘s instructions, with the libraries then sequenced using the Illumina HiSeq 2000/2500 platform (USA). As a result, we obtained an average of 20 million 100–125 bp paired reads per sample (Supplementary Table S1, S2).

### Trimming and alignment of RNA reads

The RNA-seq raw reads were cleaned, and the adapter sequences were trimmed using Trimmomatic (v0.33) (Bolger et al., 2014). Reads longer than 70 bp were retained as high-quality clean data. The purified reads acquired from the mule/hinny samples were aligned to the horse reference genome (*Equus caballus* EquCab2.0) and the donkey pseudogenome using HISAT2 (v2.0.3) (Kim et al., 2015). We constructed pseudogenomes by replacing the divergent sites without changing the genome coordinates using the method described by Wang et al. (2013). Because the genome and pseudogenome had the same genome coordinates, their mapping results were merged to eliminate mapping bias. Similarly, the purified reads acquired from the dzo samples were aligned to the cattle reference genome (*Bos taurus* Bos_taurus_UMD_3.1.1) and yak pseudogenome. To improve the mapping ratio, unmapped reads were extracted and further aligned to the corresponding genome using TopHat2 (v2.1.1) (Trapnell et al., 2009) with at most five tolerated mismatches. Reads uniquely mapped to both the genome and pseudogenome were merged for further analysis.

### Identification of fixed divergent sites and pseudogenome construction

Orlando et al*.* (2013) identified 22.6 million divergent sites between horses and donkeys (homozygous in both horse and donkey but different between horse and donkey). Divergent sites between cattle and yak were obtained through *de novo* calling. In brief, resequencing data previously obtained from six yaks (97.4G) (Qiuet al., 2012) were downloaded from the National Center for Biotechnology Information database (NCBI accession Nos.: SRR1047220, SRR1047221, SRR962824, SRR962825, SRR962826, and SRR962827) and mapped to the cattle genome using Burrows-Wheeler Alignment (BWA) (v0.7.10-r789) (Li & Durbin, 2009). Divergent site calling was then performed using the Genome Analysis ToolKit (GATK) (v3.2-2), and low-quality sites were filtered using QUAL<30.0 as a cutoff. We filtered heterozygote sites in the cattle or yak that diverged approximately 4.9 million years ago. Multiple allelic sites, including GA and GT, were also filtered to avoid inaccuracy. Finally, 20.8 million divergent sites between cattle and yak were used for analysis (Figure 1). We mapped the RNA-seq reads to the pseudogenome because mapping RNA-seq reads from hybrids to only the reference genome using the same cut-off for both reads (read arising from the reference and that from alternative alleles) can create genome mapping bias toward the reference allele. To avoid mapping bias in the hybrid transcriptome, the donkey pseudogenome was constructed by replacing the horse genome divergent sites with donkey sites using the method described by Wanget al. (2013). In brief, the donkey pseudogenome was constructed by incorporating the single nucleotide variants (SNVs) into the horse genome using the vcf2diploid tool (v0.2.6) in the AlleleSeq pipeline. We selected three horse, three donkey, and three hybrid transcriptomes, which were then mapped to the horse and donkey as well as the donkey pseudogenome to evaluate the mapping rate. Results showed that our method avoided mapping bias (Supplementary Table S3). The yak pseudogenome was constructed using the same approach. In addition, the divergent sites in the exonic region were further filtered through the cattle and yak RNA-seq data (two liver and four ear samples from cattle, two liver and two ear samples from yaks), and the divergent sites between horses and donkeys were similarly filtered using two brain, two muscle, and two skin tissue samples from donkeys and three pooled tissue samples from horses (accession numbers: ERR593552, ERR593553, and ERR593554). In addition, 647 247 and 512 588 fixed divergent sites (FDSs) located in the exonic region were used to identify the genes showing ASE and ASS of mule/hinny and dzo, respectively.

### Assignment of genetic origin of reads uniquely mapped to hybrid transcriptomes

Reads uniquely mapped to each hybrid transcriptome were assigned a genetic allele origin based on the FDSs (Figure 1). Using the dzo samples as an example, the number of cattle and yak allelic sites in all uniquely mapped paired-end reads was calculated. If the paired-end reads contained only cattle or yak allelic sites, they were regarded as being expressed from one cattle or yak allele, respectively. The paired-end reads containing both cattle and yak allele sites or those without FDSs were not included in subsequent analyses (Figure 1). Finally, 23%–42% of uniquely mapped reads from all hybrid samples were accurately assigned a genetic allele origin (Figure 2A, B; Supplementary Table S1, 2). To assess the accuracy of this result, mock hybrid transcriptomes were constructed by mixing the same amount of reads from cattle and yak (50 million). The reads of the mock hybrid transcriptomes were precisely assigned using the above-described methods (Figure 2C), and the samples of each hybrid were divided into two genetic allelic samples for ASS analysis.

### Analysis of genetic allele-specific alternative splicing

The separated genetic allelic samples were used for the detection of ASS events (Figure 1). A replicate multivariate analysis of transcript splicing (rMATS, v3.2.5)(Shen et al., 2014) was performed for the identification and comparison of gene alternative splicing events, including exon skipping (SE), mutually exclusive exons (MXEs), alternative 5' splice sites (A5SSs), alternative 3' splice sites (A3SSs), and retained introns (RIs). The likelihood-ratio method was used to test the significance of rMATS by calculating the *P* value based on the differential *ψ* values, also known as “percent spliced-in” (PSI). To ensure high-accuracy detection of ASS events, the splicing events were supported by at least 100 reads, with rigorous statistical criteria (i.e., |Δ*ψ*|>10% and false discovery rate (FDR) ≤1%) used to quantify the ASS events.

### Analysis of genes showing genetic allele-specific expression

Gene expression levels (fragments per kilobase of transcript (FPKMs)) were quantified using StringTie (v1.2.2) coupled with the R (v3.5.1) package Ballgown (v2.12.0) based on the known set of transcripts: horse, GCF_000002305.2_ EquCab2.0_genomic.gff; cattle, GCF_000003055.6_Bos_ taurus_UMD_3.1.1_genomic.gff (Pertea et al., 2016). Genes showing ASE were detected by comparing the read counts of two genetic alleles. Analysis of ASE revealed that the paternal allele was more highly expressed than the maternal allele, indicating male dominance in ASE in certain tissues. Male dominance in ASE results from an imprinting effect. In this study, we identified genetic ASE in reciprocal cross hybrids to exclude the effect of imprinting. To overcome the mapping bias of the reads, allelic expression ratios were calculated using the average read counts from the pseudogenome and reference genome. To improve the accuracy of analysis, the allelic expression ratios of each gene were calculated by combining all FDSs in the gene. In addition, those genes showing ASE were filtered under certain criteria (i.e., at least three FDSs in the exonic regions and at least 20 reads, on average, in each biological replicate). The resulting genes were used for the calculation of allelic expression ratios. In this study, the statistical significance of genes showing ASE was calculated using the Storer-Kim test (Storer & Kim, 1990). For the mule/hinny genes showing ASE, p1 and p2 were defined as the expression ratios from the horse allele and donkey allele, respectively. The expressed genes with balanced alleles showed the following expression ratio: p1=p2=0.5. We expected the genes showing ASE to have expression ratios of p1=0 and p2=1 or p1=1 and p2=0. The null hypothesis p2-p1=0 was tested, and the *P* values were corrected using the R package “qvalue” with the Benjamini-Hochberg algorithm. An adjusted *P* value of <0.05 was used. Here, to identify genes showing the most allelic imbalance, we used cutoff ratios based on previous study (Wang, 2013), that is, p1>0.65 and p2<0.35 for horse allele-specific expression and p1<0.35 and p2>0.65 for donkey allele-specific expression.

### Calculation of diversity of gene expression levels

Gene expression diversity can be reflected by the coefficient of variation (CV) of gene expression levels in biological replicates (Bellucci et al., 2014). The CV value was calculated as the ratio between the standard deviation (*SD*) and mean of gene expression levels (FPKMs) obtained for hybrid individuals. The gene expression diversity of each tissue (five brain, seven muscle, seven skin, four liver, and six ear tissue samples) was calculated separately.

### Calculation of Ka/Ks value

The coding sequence (CDS) of the horse was downloaded from ensembl (ftp://ftp.ensembl.org/pub/release-88/fasta/equus_caballus/cds/) and aligned to the donkey pseudogenome using BLAT (v36x1) with the output file type set to axt. Each alignment block in an axt file contains a summary line and two sequence lines. The summary line contains chromosomal position and size information about the alignment (details inhttps://genome.ucsc.edu/goldenPath/help/axt.html). We used the axt file to calculate the Ka/Ks value using the KaKs calculator (v2.0) (Zhang et al., 2006) with “-m GMYcN” as a parameter. Similarly, the CDS of cattle was downloaded (ftp://ftp.ensembl.org/pub/release-94/fasta/bos_taurus/cds/) and then aligned to the yak pseudogenome and then treated in the same way as above.

### Data archiving

The RNA-seq data obtained in this study were submitted to the National Center for Biotechnology Information (https://www.ncbi.nlm.nih.gov/) under accession Nos. PRJNA387435 and PRJNA387436.The RNA-seq data were also deposited at GSA (http://gsa.big.ac.cn/) under accession No. CRA001591.

## RESULTS

### Identification of genes showing allele-specific expression in hybrids

**Figure 3:**
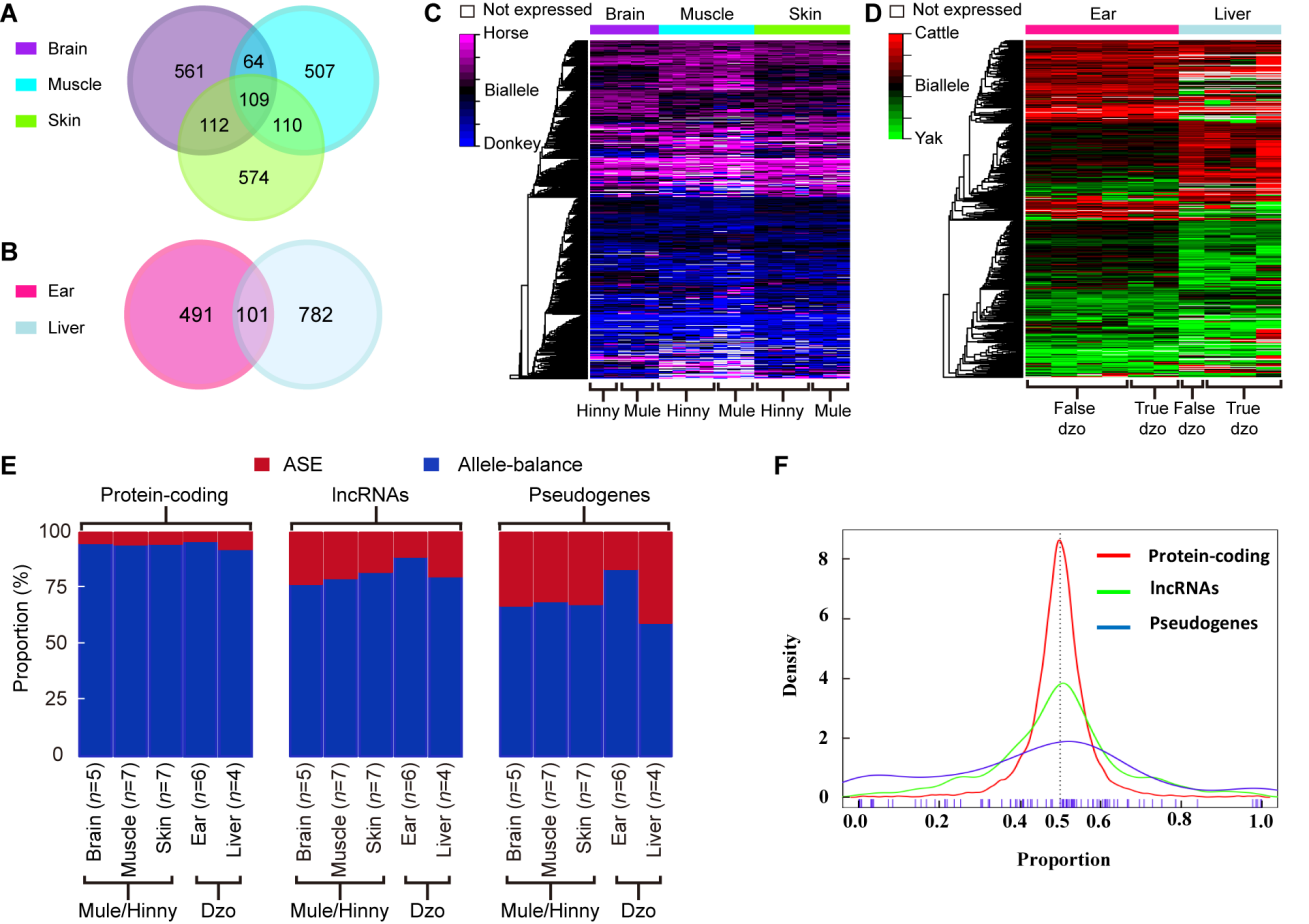
Genes showing ASE in mule/hinny and dzo Number of shared and unique genes showing ASE in brain, muscle, and skin tissues of mule/hinny (A), and ear and liver tissues of dzo (B). Heatmap of genes showing ASE (*n*=2 037) in brain, muscle, and skin samples of mule/hinny (C), and ear and liver samples of dzo (D). Genes are colored based on ASE score (red-green scale from 100%–0% horse and cattle allele expression). Side color bar shows tissues. E: Proportion of protein-coding genes, lncRNAs, and pseudogenes showing ASE and allele-balanced expression in all tested tissues of mule/hinny and dzo. Numbers of samples from each tissue are provided. F: Distribution of allelic expression ratios of protein-coding genes, lncRNAs, and pseudogenes.

To depict the *cis*-regulatory gene expression profiles, we performed RNA-seq analysis of reciprocal-cross hybrids of donkey × horse and cattle×yak (Figure 2D, E). We developed a pipeline to detect genes showing ASE (Figure 1). Based on the exonic FDSs, approximately 76.8% and 67.4% of expressed genes (13 643 of 17 770 in mule/hinny and 10 378 of 15 399 in dzo) harbored at least three FDSs in the exonic region, which enabled robust calculation of ASE (Supplementary Figure S1). To ensure precision, those genes showing ASE were filtered under the conditions that multiple FDSs (≥3) and high allelic bias (0.65/0.35) were supported by at least an average of 20 uniquely mapped reads from each sample, with concordance between biological replicates. The expression divergence between the two genetic alleles was greater than the divergence between paternal and maternal alleles (Figure 2F, G). Read number differences between paternal and maternal alleles were also compared. Here, we identified 49 imprinting genes in the mules/hinnies and 40 imprinting genes in the dzos (Supplementary Figure S2) with the same cutoff as ASE genes. Among the expressed genes (FPKM≥1), 846 (6.5%), 790 (7.3%), and 905 (6.8%) genes showing ASE were identified in the brain, muscle, and skin tissues of mule/hinny, respectively (Figure 3A, B), whereas 883 (8.7%) and 592 (4.8%) genes showing ASE were identified in the liver and ear of dzo, respectively (Figure 3A, B). When we adopted a more permissive threshold of imbalance (0.4–0.6), about 15% of genes showed ASE. Only 109 and 101 genes showing ASE were shared among the three tissues of mule/hinny and two tissues of dzo, respectively (Figure 3A). The results suggest that the genes showing ASE were tissue specific. Principal component analysis was conducted based on the allelic expression ratios (ratios of reads of two alleles), which showed that samples were clustered according to tissue type, further indicating tissue specificity in genes showing ASE (Supplementary Figure S3A, B). For example, in mule/hinny,* HPSG2* was identified as a brain-specific ASE gene (Supplementary Figure S3C) and *ST3GAL1* was identified as a muscle-specific ASE gene (Supplementary Figure S3D).

lncRNAs and pseudogenes are more likely to show ASE

**Figure 4:**
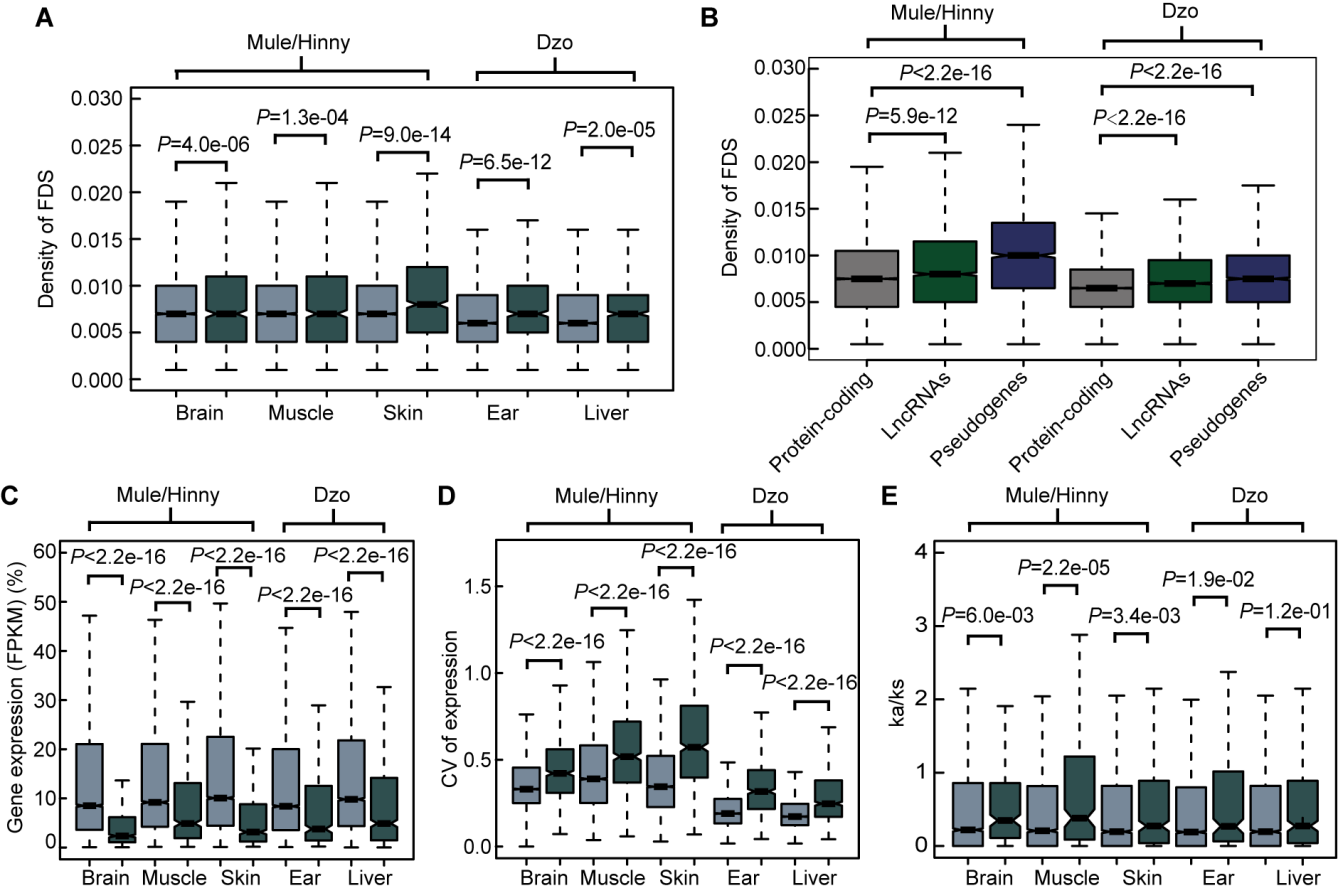
Features of genes showing ASE A: Densities of FDSs in promoter (2 kb upstream of TSS) of genes showing ASE and genes showing allele-balanced expression. B: Densities of FDSs in promoter of protein-coding genes, lncRNAs, and pseudogenes. C: Distribution of expression levels of genes showing ASE and genes showing allele-balanced expression in all tested tissues. D: Distribution of expression diversities (CV) of genes showing ASE and genes showing allele-balanced expression in all tested tissues. CV: Coefficient of variation of gene expression level in biological replicates. E: Distribution of Ka/Ks values of genes showing ASE and genes showing allele-balanced expression. Genes showing ASE are in dark-gray and genes showing allele-balanced expression are in light-gray. *P*-value was from Wilcoxon test.

Comparison of the proportions of ASE among the expressed protein-coding genes, lncRNAs, and pseudogenes showed 3.2%–6.7%, 17.3%–22.7%, and 16%–40%, respectively (Figure 3E). In addition, the distribution of allelic expression ratios further indicated that more lncRNAs and pseudogenes tended to show genetic allele biases (Figure 3F). In contrast, analysis of genes showing allele-balanced expression revealed that those genes showing ASE exhibited a higher density of divergent sites in the promoter region (Figure 4A). Consistently, a significantly higher density of divergent sites in the promoter region was observed among the lncRNAs and pseudogenes compared with the protein-coding genes (Figure 4A), which was in accordance with the proportion of ASE in these three gene types (Figure 3E). Thus, the higher proportion of divergent sites in the promoter region of lncRNAs and pseudogenes may be correlated with the evolution of gene expression. In addition, among the closely related species, the expression levels of lncRNAs and pseudogenes showed more rapid changes than that of protein coding genes.

### Changes in gene expression due to a lack of selection pressure

We subsequently searched for evidence showing the evolution of gene expression in closely related species under natural selection. First, the gene expression levels of genes showing ASE and allele-balanced expression were compared. As shown in Figure 4B, the expression levels of genes showing ASE were significantly lower than those showing allele-balanced expression in all tested tissues from both mule/hinny and dzo. This suggested that genes with low expression levels in hybrid tissues were more likely to show ASE. In contrast to the gene expression levels, the diversity in expression levels (CV, coefficient of variation of gene expression levels in biological replicates) of genes showing ASE was significantly higher than that of genes showing allele-balanced expression (Figure 4C). This high diversity indicated that the expression of genes showing ASE was not fully restricted, thus suggesting that the change in expression in most genes may have been achieved in the absence of selection pressure. To further explore the evolution of gene expression, the selection pressure (Ka/Ks ratio) of genes was investigated. A slightly higher Ka/Ks ratio was found for genes showing ASE than for genes showing allele-balanced expression; however, the values of these ratios were usually lower than 1 (Figure 4E), indicating that some genes showing ASE were under relaxed selection.

### Genes showing ASS are potential contributors to species evolution

Here, ASS events were identified using the hybrid transcriptomes with an assigned genetic allele origin through rMATS (Figure 1). To achieve relatively high-accuracy detection of ASS events, rigorous statistical criteria (|Δ*ψ*|>10%, FDR≤1%, support reads numbers ≥100) were used to quantify ASS events. In total, 980 (13.5%), 778 (12.5%), and 839 (11.2%) ASS events were identified in the brain, muscle, and skin tissues of mule/hinny (Table 1), respectively, and 559 (16.7%) and 623 (10.8%) ASS events were identified in the liver and ear of dzo, respectively (Table 1).

**Table 1:** ASS events in mule/hinny and dzo

Species	Tissue	Event type	SE	RI	MXE	A3SS	A5SS	Total
Mule/Hinny	Brain	Total splicing events	4849	151	1246	692	337	7275
		ASS events	612	23	198	75	72	980
		ASS ratio	0.126	0.152	0.159	0.108	0.214	0.135
	Muscle	Total splicing events	3992	129	1406	460	262	6249
		ASS events	458	19	193	52	56	778
		ASS ratio	0.115	0.147	0.137	0.113	0.214	0.125
	Skin	Total splicing events	4857	196	1401	660	351	7465
		ASS events	489	31	190	72	57	839
		ASS ratio	0.101	0.158	0.136	0.109	0.162	0.112
Dzo	Liver	Total splicing events	2078	108	555	423	191	3355
		ASS events	308	23	106	76	46	559
		ASS ratio	0.148	0.213	0.191	0.180	0.241	0.167
	Ear	Total splicing events	3726	203	922	616	293	5760
		ASS events	352	47	112	59	53	623
		ASS ratio	0.094	0.232	0.121	0.096	0.181	0.108

**Figure 5:**
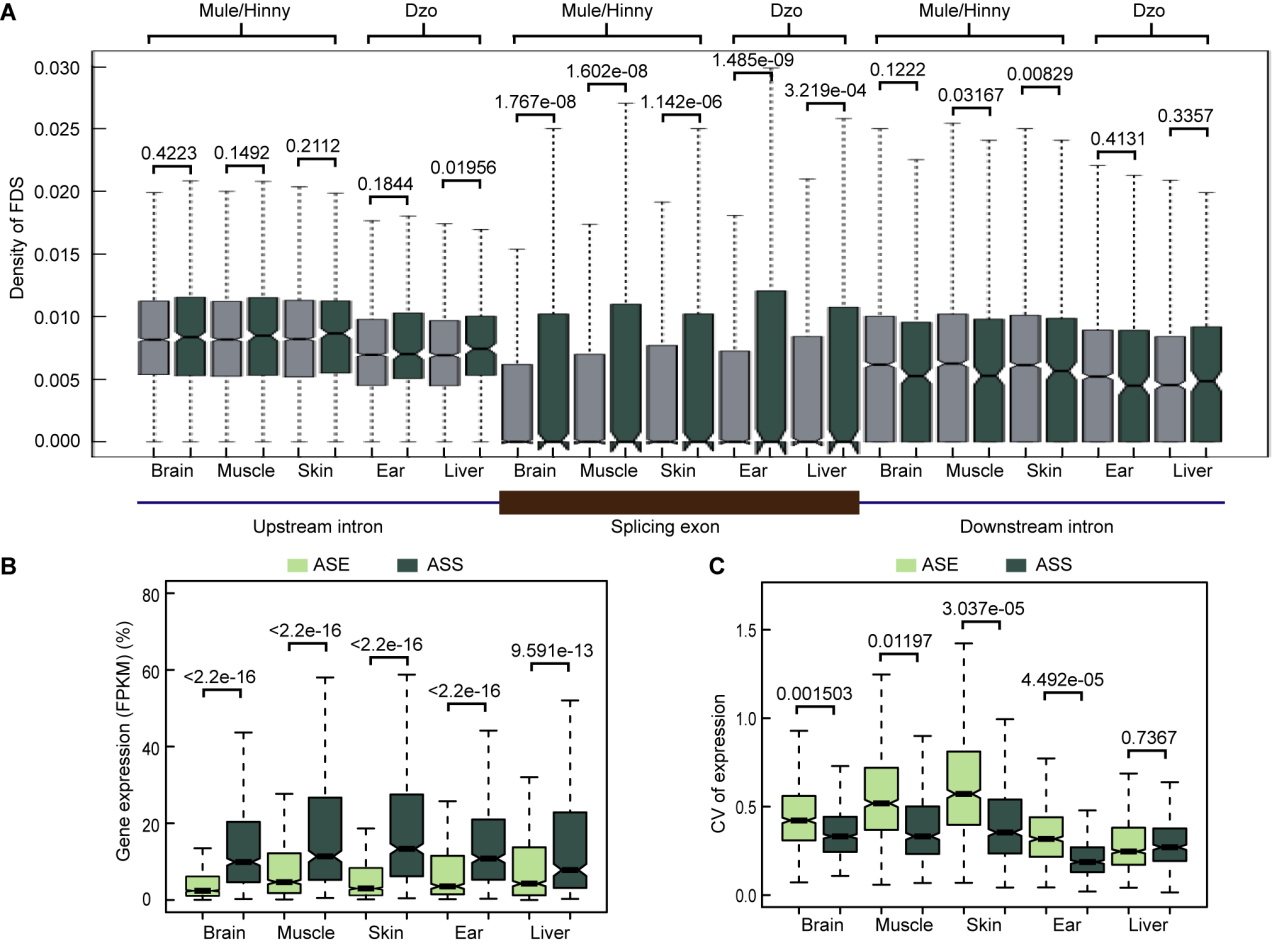
Features of genes showing ASS A: Densities of FDSs in exons, upstream introns, and downstream introns of ASS and non-ASS events (Wilcoxon test). B: Comparison of expression levels of genes showing ASE and genes showing ASS. C: Comparison of expression diversities (comparison of genes) of genes showing ASE and genes showing ASS.

We detected the density of FDSs in the splicing exons and adjacent introns of ASS and non-ASS events. As shown in Figure 5A, compared with non-ASS exons, the densities of FDSs in the ASS exons were higher, whereas the densities in upstream and downstream introns showed no differences. These results suggest that the FDSs within the exons may have led to gene splicing evolution. In addition, genes showing ASS had higher expression levels than genes showing ASE in all tested tissues (Figure 5B). In contrast, the diversity in the expression levels of genes showing ASS was lower that of genes showing ASE in all tissues, with exception of the dzo liver (Figure 5C). Thus, the evolution of *cis*-regulatory alternative splicing may be a major contributor to the adaptive evolution of a species and may be equally important to the evolution of gene expression levels.

### Genes showing ASE and ASS are involved in species adaptive evolution

**Figure 6:**
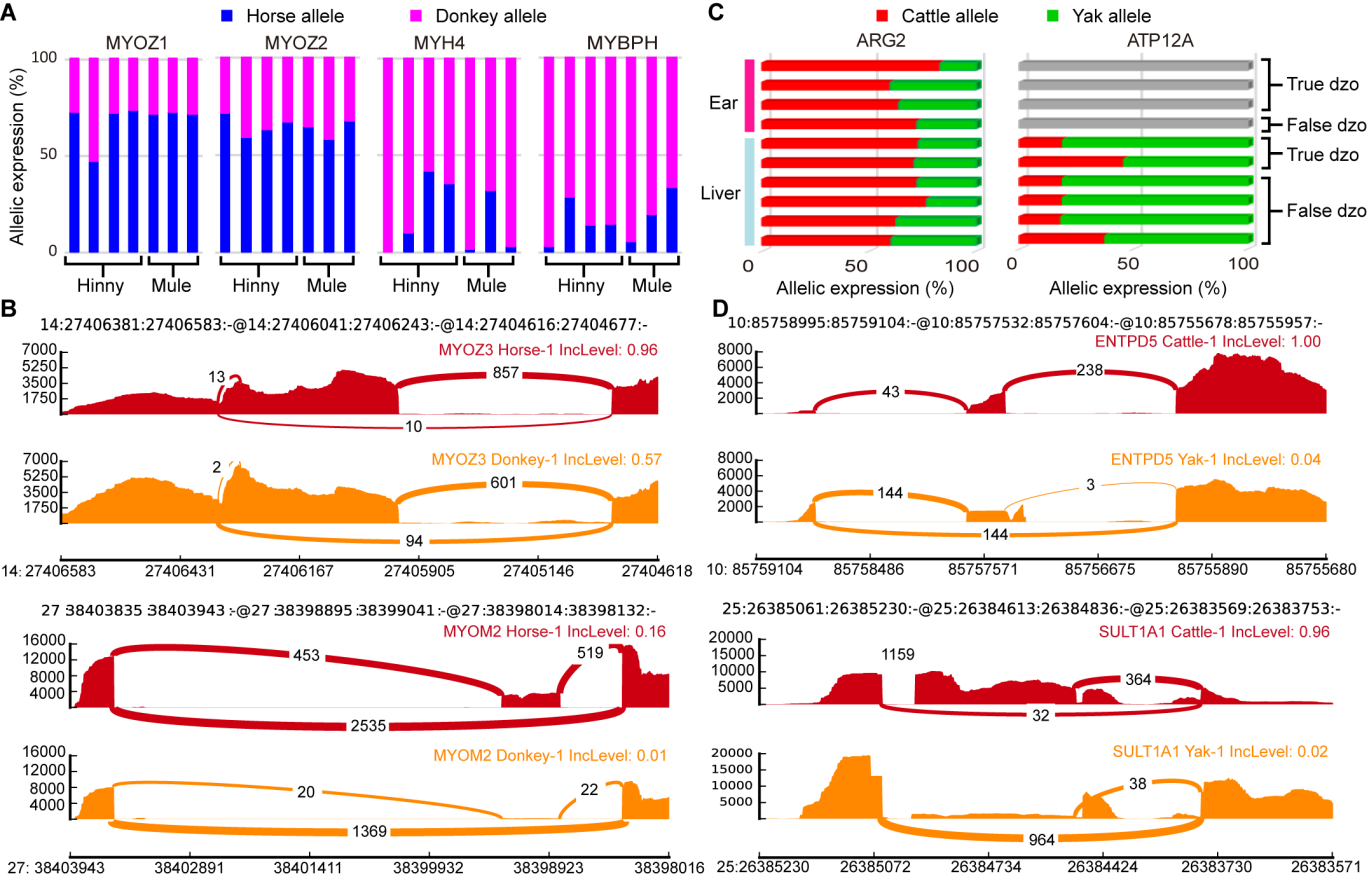
Functional analysis of genes showing ASE and ASS A: Allelic expression ratios of four muscle function-related ASE genes, *MYOZ1*, *MYOZ2*, *MYH4*, and *MYBPH*, in mule/hinny muscle samples. Horse allele (blue); donkey allele (purple). B: Sashimi plot of muscle-related genes showing ASS, *MYOZ3*, and *MYOM2*, in mule/hinny muscle samples. Read densities supporting inclusion and exclusion of exons are shown. C: Proportion of *ARG2* and *ATP12A* expression levels from cattle allele (red) or yak allele (green) in four liver and six ears samples of dzo. Gray bar indicates no expression. D: Sashimi plot of two genes showing ASS (*ENTPD5 *and *SULT1A1*) in dzo. Read densities supporting inclusion and exclusion of exons are shown.

Genes showing ASE and ASS in mule/hinny muscle tissue have received considerable attention since the discovery of muscle strength divergence between horses and donkeys (Renaud et al., 2018). Here, four genes showing ASE (i.e., *MYOZ1*, *MYOZ2*, *MYH4*, and *MYBPH*)were detected in all mule/hinny muscle samples (Figure 6A). Notably, *MYOZ1* and *MYOZ2* are members of the same *MYO* gene family, which plays a role in myofibrillogenesis (Takada et al., 2001), with both found to be donkey specific in the current study. In contrast, however, both MYH4 and MYBPH were found to be horse specific (Figure 6A). Thus, these mule/hinny muscle-related genes showing ASE may be responsible for the divergence observed in muscle strength between horses and donkeys. Furthermore, *MYOZ3* and *MYOM2* showed ASS in the mule/hinny muscle samples (Figure 6B). Notably, *MYOZ3* also belongs to the *MYO* gene family, suggesting that the *MYO* gene family may have played an important role in the evolution of muscle strength. Thus, the above results further suggest that changes in both gene expression levels and alternative splicing have played a role in the divergence of muscle strength found between horses and donkeys.

Compared with cattle, yaks exhibit a remarkable adaptive trait to high-altitude hypoxic environments. In the current study, the *ARG2* gene showing ASE was identified in both the liver and ear of dzo (Figure 6C). *ARG2* has been identified previously as a hypoxia-related and rapidly evolving gene in yak (Qiu, 2012). In addition, the *ATP12A* gene showing ASE was identified with high expression in the yak allele of dzo (Figure 6C). This gene has been identified previously as a hypoxia-related gene in Tibetan antelope (Ge et al., 2013). In addition, we also identified *ENTPD5* and *SULT1A1* as dzo genes showing ASS (Figure 6D). These two genes are involved in metabolic processes (Gamage et al., 2006; Huitema et al., 2012), and thus may also contribute to the high-altitude adaptation of yak. Thus, the above results provide preliminary evidence demonstrating that genes showing ASE and ASS may participate in the regulation of muscle strength in mule/hinny and high-altitude adaptation in dzo.

## DISCUSSION

To date, most previous studies on genes showing ASE have been conducted on model organisms, e.g., mouse (Crowley, 2015; Gao, 2015). As their hybrids can be difficult to obtain, few studies have been reported on genes showing ASE in large mammals, particularly in regard to changes in gene expression in animal speciation and evolution. In this study, we obtained the ASE and ASS genetic profiles of horse×donkey and cattle×yak hybrids to reveal differences in gene expression and alternative splicing between closely related species. The use of closely related species with more FDSs allows for the robust and precise identification of genes showing ASE and ASS.

Protein-coding genes and lncRNAs are known to contribute to species evolution (Babbitt et al., 2010). However, previous study has indicated that the sequences of lncRNAs change more rapidly than those of protein-coding genes (Johnsson et al., 2014). For example, in humans, one of the most rapidly evolved regions (“human accelerated regions”) from chimpanzees, i.e., HAR1, is a noncoding RNA gene (Pollard et al., 2006). Compared with protein-coding genes, we found that lncRNAs were more likely to show ASE. Hence, our results indicated that the expression levels of lncRNAs were also rapidly evolving between closely related species. Similar to lncRNAs, pseudogenes, which are regarded as dysfunctional DNA sequences without selective constraints and are thus silenced last (Mira, 2005), also tended to show ASE. Some detected pseudogenes showing ASE may be in the process of elimination, i.e., are expressed in one species but not in another, thus resulting in ASE. As such, most gene expression changes (e.g., ASE) may be neutral and not under evolutionary selection. However, some gene expression changes may contribute to adaptive evolution; for example, the ASE genes (*MYOZ1*, *MYOZ2*, *MYH4*, and *MYBPH*) involved in the regulation of muscle strength in mule/hinny. These results are coincident with most genetic changes being neutral, with only a few being under positive selection. Thus, while many ASE genes may be evolutionary neutral, some may be related to the adaptive evolution of a species. Here, in each tested mule/hinny and dzo tissue using cutoff ratios of below 0.35 and above 0.65 (Wang, 2013), approximately 5% of expressed genes were identified as showing ASE. However, previous research on mice (Pinter, 2015) and *Drosophila* (León-Novelo et al., 2017) observed 20% ASE genes. We thus applied a more permissive threshold of imbalance (0.4–0.6), with results indicating that about 15% of genes showed ASE. However, to be consistent with the pipeline in Wang et al. (2013), we still used the gene list with the cutoff of 0.35–0.65. Based on this, our results suggested that most *cis*-regulatory effects on gene expression did not differ between closely related species. This is consistent with the view that gene expression evolution is conserved and strongly shaped by purifying selection (Jordan, 2004; Liao & Zhang, 2006; Zheng-Bradley et al., 2010). In addition, genes showing ASE had lower expression levels, higher expression diversity, and slightly higher Ka/Ks ratios than genes showing allele-balanced expression. These results indicate that the changes in the expression levels of most genes may have occurred in the absence of selection pressure. Gene alternative splicing is actually more prevalent than previously anticipated, with more than 90% of human genes possessing different transcription isoforms (Wang et al., 2008). We discovered that more than 10% of alternative splicing events showed significant differences between related species, which was higher than that found for ASE. In addition, the expression levels of genes showing ASS were higher than those showing ASE, whereas the diversity in the expression of genes showing ASS was lower than that for genes showing ASE. In conclusion, our study demonstrated that both gene expression and alternative splicing contribute to the divergence between closely related species.
